# Social Media Dynamics and Green Consumption—The Mediating Role of Environmental Attitudes and Green Self-Identity—Cross-Country Research

**DOI:** 10.3390/foods15132408

**Published:** 2026-07-07

**Authors:** Jorge Bernal-Peralta, Nelson Carrión-Bósquez, Wilson Zambrano-Vélez, Mirella Correa-Peralta, Mario Vidal-Alfaro, Ninfa Willans-Muñoz, Rubén Marchena-Chanduvi, Andrés Vélez-Luna, Ignacio López-Pastén, Mary Llamo-Burga

**Affiliations:** 1 Facultad de Administración y Economía, Universidad de Tarapacá, Arica 1000007, Chile; 2 Departamento de Administración, Facultad de Economía y Administración, Universidad Católica del Norte, Antofagasta 1270709, Chile; 3 Facultad de Ciencias Sociales y de la Salud, Universidad Estatal Península de Santa Elena, La Libertad 240250, Ecuador; wzambrano@upse.edu.ec; 4 Facultad de Ciencias e Ingeniería, Universidad Estatal de Milagro, Milagro 091050, Ecuador; mcorreap@unemi.edu.ec; 5 Departamento de Ingeniería Comercial, Facultad de Ingeniería, Universidad de Antofagasta, Antofagasta 1270300, Chile; mario.vidal@uantof.cl (M.V.-A.); ninfa.willans@uantof.cl (N.W.-M.); 6 Escuela Profesional de Ingeniería Agroindustrial, Facultad de Ciencias Agrarias, Universidad Nacional Autónoma de Chota, Cajamarca 06001, Peru; rmarchenac@unach.edu.pe; 7 Extensión El Carmen, Facultad de Ciencias Administrativas, Contables y Comercio, Universidad Laica Eloy Alfaro de Manabí (ULEAM), El Carmen 130450, Ecuador; andres.velez@uleam.edu.ec; 8 Facultad de Ingeniería y Negocios, Universidad Santo Tomás, Antofagasta 1240000, Chile; ilopez7@santotomas.cl; 9 Escuela Profesional de Ingeniería en Agronegocios, Universidad Nacional de Cajamarca, Cajamarca 06001, Peru; mllamob@unc.edu.pe

**Keywords:** social media content, online member group support, environmental attitude, green self-identity, organic food purchasing behavior, multigroup analysis

## Abstract

This study examined the influence of social media content and online member group support on the environmental attitudes and green self-identity of organic food consumers in Ecuador, Peru, and Chile. Based on the Stimulus–Organism–Response (SOR) framework, data were collected from 766 consumers in Ecuador (n = 310), Peru (n = 259), and Chile (n = 197) through an online survey, the participants were adults from Ecuador, Peru, and Chile with different educational backgrounds who had purchased or consumed organic products during the month preceding the survey. The proposed model was assessed using partial least-squares structural equation modeling. The results revealed that both social media content and online member group support positively influenced environmental attitudes, while environmental attitudes significantly strengthened green self-identity, which in turn positively affected purchasing behavior of organic products. Although some relationships varied across countries, the mediating effects of environmental attitudes were consistently supported. Furthermore, the Measurement Invariance of Composite Models procedure established compositional invariance for all constructs across the three country pairs, and multigroup analysis did not identify significant differences in structural relationships between Ecuador and Peru or between Ecuador and Chile. These findings confirm the transnational robustness of the proposed framework, providing valuable insights into how digital social environments influence environmental attitudes, strengthen ecological self-identity, and promote the purchase of organic foods.

## 1. Introduction

The sustained increase in greenhouse gas emissions, biodiversity loss, pollution of terrestrial and marine ecosystems, and the progressive depletion of natural resources have been widely associated with resource-intensive production and consumption patterns, creating unprecedented pressure on socio-ecological systems [1,2]. In this context, mitigating anthropogenic climate change is no longer conceived solely as the responsibility of governments and productive organizations but now actively incorporates the role of the consumer as an agent of transformation [3,4,5,6]. From this perspective, purchasing decisions represent everyday mechanisms through which individuals can reduce their ecological footprint and contribute to the transition toward more sustainable development models [7,8,9]. Among the various expressions of responsible consumption, the Purchasing Behavior of Organic Products has gained particular relevance because of its association with less polluting agricultural practices, reduced use of synthetic chemical inputs, and more sustainable management of natural resources [10,11]. In recent years, the organic food sector has experienced sustained growth across Latin America, driven by increasing consumer awareness of health, environmental sustainability, and food quality. Ecuador and Peru have consolidated their positions as leading exporters of organic products [1,2], whereas Chile has strengthened both its domestic organic market and sustainability-oriented food systems through the expansion of certified organic production and growing consumer demand [12]. These developments reflect a regional transition toward more sustainable food consumption patterns, while simultaneously highlighting the importance of understanding the psychological and digital factors that encourage consumers to purchase organic products within comparable Latin American contexts. In this sense, understanding the factors that explain the effective adoption of this behavior is a theoretical and practical priority to promote consumption patterns compatible with global sustainability goals.

Understanding environmentally responsible purchasing behavior requires examining the internal mechanisms through which individuals integrate pro-environmental behaviors into their self-concept [13,14,15]. In this regard, Green Self-Identity (GSI) has emerged as a key construct for explaining why some consumers consistently engage in environmentally responsible behaviors over time [16]. Specifically, GSI reflects the extent to which individuals perceive themselves as committed to environmental protection and adopting sustainable lifestyles [17]. However, the development of a strong ecological identity is often preceded by evaluative processes that shape favorable dispositions toward environmental protection [18,19,20]. Within this context, Environmental Attitude (EAT) has been recognized as one of the most extensively studied constructs to explain the adoption of sustainable behaviors [21,22].

EAT is conceptually defined as the cognitive, affective, and evaluative judgment that individuals hold regarding environmental issues, preservation of natural resources, and actions intended to mitigate human impacts on the environment [23,24]. Within major behavioral theories, attitudes are regarded as proximal determinants of behavioral intentions, as they provide an interpretative lens through which individuals assess alternatives and formulate behavioral responses [25]. In the domain of sustainable consumption, a positive EA enhances consumers’ propensity to value the ecological attributes of products; perceive benefits associated with environmentally responsible choices; and exhibit a greater willingness to incur the economic, practical, or behavioral costs that such decisions may require [26,27].

The development of favorable EAT increasingly occurs within a context characterized by pervasive digital connectivity and continuous exposure to interactions mediated through social media platforms [28,29]. In this regard, social media platforms have evolved beyond their original role as communication channels to become complex sociocultural infrastructures capable of shaping perceptions, disseminating social norms, and facilitating large-scale observational learning processes [30,31]. Within this digital ecosystem, two stimuli are particularly relevant to understanding the formation of pro-environmental orientations. First, Social Media Content (SMC) functions as a predominantly informational and cognitive stimulus by providing knowledge, narratives, visual evidence, and persuasive messages related to sustainability, environmental issues, and responsible consumption alternatives [32,33,34]. Second, Online Member Group Support (OMG) operates as a primary social and affective stimulus, characterized by the exchange of experiences, interpersonal validation, a sense of belonging, and emotional support generated within virtual communities built around shared environmental values [35,36].

Despite growing scholarly interest in sustainable consumption and the role of digital environments in promoting environmentally responsible behaviors, the existing body of knowledge remains constrained by several conceptual and structural limitations. A substantial number of prior studies have examined the effects of social media [37], group interaction [38], EAT [26], self-identity [17], and green purchasing [31] as largely independent phenomena, resulting in a fragmented understanding of the mechanisms that connect these constructs. Furthermore, Yen and Hoang [39] found that online product reviews, which are considered a form of social stimulus, do not necessarily foster favorable attitudes toward environmentally friendly alternatives. Similarly, Addisu and Rhee [40] reported that consumers might respond negatively to communication-based stimuli under certain conditions. Consequently, there remains a limited understanding of how social and communicative stimuli embedded within digital environments are progressively internalized by consumers and transformed into actual sustainable purchasing behaviors.

In light of the foregoing, there remains a lack of integrated theoretical frameworks capable of explaining the sequential process through which SMC and OMGS influence EA, strengthen GSI, and ultimately lead to the adoption of Purchasing Behavior of Organic Products (PBOP). Consequently, comprehensive explanatory models that provide a deeper understanding of the psychological and social architecture underlying the transition from digital influence to environmentally responsible purchasing decisions are needed. Accordingly, the present study aims to examine how SMC and OMGS influence EAT and GSI and how these psychological mechanisms subsequently shape the PBOP among South American organic food consumers. To fulfill the proposed objective, this study provides answers to the following research questions:

- How does SMC influence the EAT and GSI of South American consumers?
- How does OMGS influence the EAT and GSI of South American consumers?
- How does EAT influence the GSI of South American consumers?
- How does EAT mediate the relationship between SMC, OMGS, and the GSI of South American consumers?
- How does the GSI influence the PBOP of South American consumers?

This study makes a relevant empirical contribution by extending the analysis to three Latin American national contexts: Ecuador, Peru, and Chile. The novelty of this study lies in distinguishing between two digital stimuli that are frequently treated as equivalent in prior research: social media content as an informational–cognitive stimulus and online member group support as a socio-affective stimulus. By integrating these stimuli into a sequential SOR-based model, this study explains not only whether digital environments influence organic food purchasing behavior but also how such influence is internalized through environmental attitudes and subsequently transformed into green self-identity. This approach advances previous research by showing that digital exposure and online community support do not automatically generate sustainable purchasing behavior; rather, their effect depends on a progressive psychological process through which consumers first favorably evaluate environmental issues and then incorporate these values into their self-concept. On the other hand, the selection of Ecuador, Peru, and Chile was theoretically motivated by the need to evaluate the robustness of the proposed SOR model in culturally related but structurally heterogeneous contexts. Although these three South American countries share historical, linguistic, and broad cultural characteristics that reduce the influence of major cultural confounders, they differ in relevant dimensions associated with sustainable consumption, including the maturity of organic food markets, diffusion of environmental policies, adoption of digital technologies, and consumer engagement with sustainability initiatives. Examining the proposed relationships across countries that are sufficiently comparable yet exhibit meaningful contextual variation provides a more rigorous assessment of the external validity of the model. Consequently, rather than seeking maximum cultural contrast, this study tests whether the psychological mechanisms linking digital social environments to sustainable purchasing behavior remain stable across comparable Latin American contexts characterized by different levels of market and institutional development.

## 2. Literature Review

### 2.1. Theoretical Framework

#### Stimulus–Organism–Response Framework

**Figure 1:**
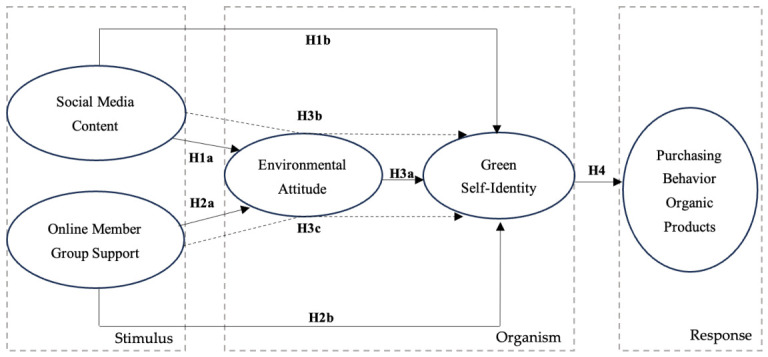
Hypothesized research model.

The present study adopts the Stimulus–Organism–Response (SOR) framework as its theoretical foundation for explaining the social mechanisms underlying PBOP. According to this framework and the hypothesized model proposed in this study (see Figure 1), SMC and OMGS represent the stimuli that influence EAT and GSI (organism), respectively, while the response is reflected in PBOP. The primary strength of the SOR framework lies in its ability to explain how environmental stimuli are internalized through cognitive, affective, and identity-related processes before manifesting as observable behavioral responses [41,42]. Owing to its strong explanatory power, the SOR framework has been widely applied to understand behaviors related to sustainable consumption, innovation adoption, digital platforms, virtual communities, and responsible purchasing decisions [43,44,45,46,47,48], establishing itself as one of the most robust conceptual frameworks for examining the interactions between digital environments and consumer behavior.

The SOR framework is preferred because it provides a more suitable structure for explaining how external digital stimuli are internalized before generating behavioral responses [31]. Although the Theory of Planned Behavior, Value–Belief–Norm Theory, Social Identity Theory, and the Norm Activation Model have made important contributions to green consumption research, they mainly emphasize intentions, moral obligations, values, norms, or identity-based processes [1,2]. In contrast, the present study specifically examines how social media content and online member group support operate as environmental stimuli that influence consumers’ internal psychological states, namely, environmental attitudes and green self-identity, before translating into organic food purchasing behavior. Therefore, SOR offers a broader explanatory architecture that integrates informational, social, and attitudinal mechanisms within a single sequential model [44]. This makes it particularly appropriate for analyzing digital social environments, where consumer behavior emerges from the interaction between external online cues and internal psychological processes.

### 2.2. Conceptual Framework

#### 2.2.1. Social Media Content

SMC represents a dynamic source of information composed of diverse communication formats, including written posts, images, videos, multimedia elements, and hyperlinks that are created, shared, and consumed on interactive digital platforms [49]. Unlike traditional one-way media channels, these platforms enable users to actively participate in the creation, interpretation, and evaluation of content, fostering bidirectional communication processes and the collective construction of meaning [50]. In the context of consumer behavior, the influence of such content largely depends on the degree of usefulness, credibility, and relevance attributed to it by individuals, as these perceptions shape how consumers interpret issues related to the environmental consequences of their consumption decisions [51]. Consequently, repeated exposure to information concerning sustainable practices may reinforce positive evaluations of environmentally responsible consumption alternatives, including organic products [52]. Likewise, such content can contribute to the development of cognitive and evaluative frameworks that support sustainability and strengthen personal convictions related to environmental protection [53]. Therefore, understanding the processes through which environmental cues transmitted in digital environments are interpreted and incorporated into consumers’ decision-making frameworks remains an important area of inquiry [54].

The existing literature has highlighted that content shared through social media serves not only an informational function but also facilitates user interactions that promote the exchange of experiences, knowledge, and resources related to common interests [55]. These dynamics reflect the defining characteristics of contemporary digital ecosystems, where the dissemination of information depends largely on the participation, recommendations, and collaborative efforts of virtual community members [56]. From this perspective, social media platforms constitute fertile environments for the creation of collective value by enabling individuals to actively engage in discussions and initiatives centered on shared objectives [57]. In particular, related emerging interactions within these spaces can generate emotional bonds and symbolic meanings that encourage greater involvement in the causes of sustainability and environmental responsibility [58]. Furthermore, social media provides effective mechanisms for coordinating collective efforts, disseminating awareness campaigns, and mobilizing actions aimed at addressing the environmental challenges of common concern [59]. Consequently, content created and shared through these platforms has become a strategic vehicle for promoting the values, knowledge, and behaviors associated with sustainable development [52].

Despite the growing academic interest in understanding the effects of SMC on consumer behavior, important theoretical limitations remain regarding the mechanisms through which digital influences translate into observable behaviors [54,60,61]. Although numerous studies have documented associations between exposure to digital content and various forms of sustainable behavior, an understanding of the psychological processes underlying this relationship remains fragmented, particularly in the context of organic product consumption [59]. Consequently, further research is needed to explain how sustainability-related messages are cognitively processed by individuals and subsequently transformed into purchasing decisions that align with environmental values and principles [53]. Based on these considerations, this study proposes the following hypotheses:

**Hypothesis 1a (H1a).**  *SMC positively influences the EAT of South American consumers.*

**Hypothesis 1b (H1b).**  *SMC positively influences the GSI of South American consumers.*

#### 2.2.2. Online Group Support

OMGS refers to the informational, emotional, and social support that individuals receive through virtual communities formed around shared interests, values, or objectives [35,36]. These communities typically develop across various digital platforms, including Facebook, Instagram, TikTok, and other online social interaction spaces, where users actively engage through communication, experience sharing, and dissemination of information relevant to the group [50,51]. Beyond facilitating access to recommendations and opinions from other participants, these interactions contribute to the construction of collective meanings and reinforcement of social identities developed within digital environments [35,36,52,62,63,64]. Consequently, participation in such communities can significantly influence how individuals interpret their surroundings, evaluate consumption alternatives, and develop specific attitudinal orientations [31]. Furthermore, the sense of belonging generated within these spaces fosters the creation of affective and normative ties that may exert a substantial influence on consumer decisions and behaviors [65].

Within the context of sustainable consumption, digital communities dedicated to the discussion and promotion of organic products have gained increasing relevance as spaces for socialization and collective learning [66,67]. Through these platforms, participants can access specialized information, share consumption experiences, exchange recommendations, and strengthen their knowledge of environmentally responsible practices [68]. Continuous interaction among members who share similar environmental concerns facilitates the consolidation of positive beliefs and evaluations regarding sustainability, while reinforcing identification with common environmental values [69,70]. In this context, the support perceived by other community members may increase consumers’ confidence in their decisions and strengthen their willingness to adopt behaviors consistent with the principles promoted by the group [31]. Consequently, these communities extend beyond their communicative function and operate as mechanisms of social influence capable of shaping perceptions, attitudes, and even behaviors related to green consumption [71].

Despite the increasingly prominent role of social media and virtual communities in shaping consumption behaviors, scientific knowledge regarding the specific impact of support received within these online groups remains limited [36]. Consequently, there is still a need to better understand whether continuous exposure to pro-environmental discourse and frequent interaction with members who endorse sustainable practices contribute to strengthening consumers’ commitment to purchasing organic products. Therefore, this study proposes the following hypotheses:

**Hypothesis 2a (H2a).**  *OMGS positively influences the EAT of South American consumers.*

**Hypothesis 2b (H2b).**  *OMGS positively influences the GSI of South American consumers.*

#### 2.2.3. Environmental Attitude

EAT represents a relatively stable psychological orientation through which individuals evaluate, interpret, and respond to issues related to environmental protection and conservation of natural resources [72,73]. This disposition integrates cognitive, affective, and evaluative components that reflect the degree of concern individuals hold regarding environmental problems and their commitment to sustainability-oriented practices [74,75,76]. Within the consumption context, EAT functions as an interpretative framework that guides the evaluation of products and market alternatives, particularly those associated with ecological benefits or sustainable attributes [77,78,79]. Consequently, consumers with a more favorable EAT tend to evaluate consumption offerings that promote environmental impact reduction and ecosystem preservation more positively [21,24,29,48]. The importance of EAT in the study of sustainable consumer behavior lies in its capacity to connect environmental awareness with concrete marketplace actions [77,78,80]. Beyond merely reflecting concern for environmental issues, this construct serves as a motivational mechanism that directs individual decisions toward alternatives consistent with sustainability values [81,82]. Consequently, consumers exhibiting higher levels of environmental sensitivity demonstrate a greater willingness to select products perceived as less harmful to the environment, even when such choices involve higher economic costs, additional search efforts, or changes in previously established consumption habits [83,84,85]. From this perspective, EAT not only influences specific purchasing decisions but also contributes to the consolidation of consumption patterns characterized by greater social and environmental responsibility [74].

Accumulated evidence consistently identifies EAT as one of the most robust predictors of the intentions and behaviors associated with the purchase of environmentally friendly products [86,87,88]. Numerous studies have reported that consumers with higher levels of environmental concern exhibit a stronger predisposition toward consumption alternatives aligned with ecological principles and sustainable practices [75]. Likewise, EAT can be strengthened through environmental education, personal experiences related to ecological issues, social interactions, and exposure to awareness campaigns designed to promote responsible behaviors [89,90]. Given that the present study sought to understand the psychological mechanisms underlying PBOP, EAT may constitute a fundamental internal state capable of transforming influences originating from the social and digital environment into concrete behavioral responses. The following hypothesis is proposed to empirically examine this proposition:

**Hypothesis 3a (H3a).**  *EAT positively influences the GSI of South American consumers.*

**Hypothesis 3b (H3b).**  *EAT mediates the relationship between SMC and South American consumers’ GSI.*

**Hypothesis 3c (H3c).**  *EAT mediates the relationship between OMGS and South American consumers’ GSI.*

#### 2.2.4. Green Self-Identity

GSI represents the extent to which individuals incorporate environmental values and attributes into their self-concept, perceive themselves as people committed to environmental protection, and adopt sustainable consumption practices [91]. In this regard, GSI goes beyond merely holding favorable perceptions of environmental issues; rather, it reflects a relatively stable representation of who individuals are and how they wish to act in response to sustainability-related challenges [92,93]. When environmental concerns are integrated into an individual’s self-concept, consumption decisions cease to be driven solely by rational evaluations or external incentives and instead become coherent expressions of personal identity [94]. Consequently, GSI can be understood as a psychological mechanism that promotes consistency between individuals’ environmental values and sustainable consumption behaviors [95].

The literature has identified GSI as one of the most influential psychological determinants of pro-environmental behaviors and purchasing decisions related to sustainable products [96]. Empirical evidence suggests that GSI strengthens the relationship between environmental beliefs and concrete actions, facilitating the translation of ecological concerns into observable responsible consumption behaviors [93,97,98]. Similarly, individuals who perceive themselves as green consumers tend to exhibit more favorable attitudes toward environmentally friendly products and a stronger propensity to maintain consumption patterns consistent with this identity over time [91,94]. Consequently, GSI has been recognized as an internal driving force capable of enhancing the persistence of sustainable behaviors and promoting purchasing decisions aligned with the principles of environmental responsibility [93,98].

Most previous studies have focused on examining the direct effects of GSI on ecological intentions and behaviors, while paying comparatively less attention to the antecedent processes that explain its formation and activation within sustainable consumption contexts [99,100,101]. Furthermore, several studies have demonstrated that GSI exerts indirect effects on purchase intentions through a variety of context-specific variables [94,102,103,104]. In light of these findings, the need to determine whether GSI emerges as a consequence of contextual influences and subsequently contributes to the development of environmentally responsible behaviors motivated the present study to test the following hypothesis:

**Hypothesis 4 (H4).**  *The GSI of South American consumers positively influences their PBOP.*

#### 2.2.5. Critical Synthesis of Previous Research and Remaining Research Gaps

Although previous research generally supports positive associations among digital communication, environmental attitudes, green self-identity, and sustainable purchasing behavior, the accumulated evidence remains fragmented and only partially integrated. Most existing studies have examined these constructs independently or have focused on isolated relationships, such as the influence of social media on environmental awareness, the role of environmental attitudes in sustainable consumption, and the effect of green self-identity on purchasing decisions, without simultaneously explaining the complete psychological process linking digital environments with actual purchasing behavior [17,26,31,36,52,59].

Furthermore, the empirical evidence is not entirely consistent. While several studies have reported that sustainability-oriented SMCs contribute to strengthening EAT and encouraging environmentally responsible purchasing [52,53,57], other investigations suggest that digital exposure alone is insufficient to produce enduring behavioral change or identity transformation [39,40,54,60,61]. Similarly, research examining OMGS has yielded mixed findings. Some authors argue that virtual communities facilitate social learning, environmental engagement, and pro-environmental behaviors through emotional support and shared experiences [35,36,63,65], whereas others indicate that these effects may be weak, indirect, or highly dependent on contextual and individual conditions [31,38]. Similar inconsistencies were also evident in the relationship between the EAT and GSI. Although EAT is consistently recognized as an important antecedent of sustainable behavior [21,74,77], there is considerably less agreement regarding the psychological mechanisms through which these attitudes evolve into a stable ecological self-concept [91,94,99].

Taken together, these inconsistencies indicate that current knowledge remains limited in explaining how different forms of digital influence are sequentially internalized before becoming an observable, sustainable purchasing behavior. Accordingly, this study addresses this gap by proposing an integrated SOR-based model in which SMC and OMGS are conceptualized as complementary digital stimuli that influence purchasing behavior through two interconnected psychological mechanisms: EAT and GSI.

### 2.3. Research Model

In order to fulfill the objective proposed in the research, the model to be hypothesized is presented below (See Figure 1).

## 3. Materials and Methods

### 3.1. Instrument Design and Data Collection

The present study was conducted under a positivist paradigm, which posits that theoretically proposed relationships can be empirically tested through systematic collection and analysis of quantitative evidence. Consistent with this epistemological perspective, the study adopted a deductive research strategy to empirically evaluate the hypotheses derived from previously established theoretical foundations, particularly the SOR framework. From a methodological standpoint, the study was structured using a quantitative, cross-sectional, and explanatory research design, aimed at examining the relationships among the constructs proposed in the conceptual framework at a specific point in time.

Data were collected using a structured questionnaire comprising three sections. The first section presented the informed consent form, which explained the objectives of the study and the conditions governing the participants’ voluntary involvement. The second section was designed to collect sociodemographic information using five descriptive questions: age, gender, nationality, educational level, and country of residence. Finally, the third section included the items used to measure the study variables, comprising a total of 20 indicators distributed across the five constructs included in the research model (SMC, OMGS, EAT, GSI, and PBOP). Prior to data collection, the instrument underwent content validation through expert judgment to assess the clarity, relevance, and conceptual consistency of each item. Subsequently, a pilot study involving 30 participants was conducted to verify item comprehension, identify potential ambiguities, and confirm the suitability of the questionnaire for the study context. No comments were made by the participants in the pilot test regarding the clarity and understanding of the questions.

The measurement instruments were adapted from previously validated scales and adjusted according to the specific characteristics of the present study. All constructs were measured using seven-point Likert scales. Specifically, the items corresponding to SMC were adapted from Li et al. [105], the items measuring OMGS were adopted from Shihab et al. [36], the measures of EAT and PBOP were adapted from Carrión et al. [77], and the items assessing GSI were adapted from Confente et al. [95] (See Appendix A). The participants were selected using a non-probability convenience sampling procedure. The target population consisted of adults residing in Ecuador, Peru, and Chile who had purchased or consumed organic products during the month preceding the survey. Participants represented different educational levels, including secondary education, technical education, undergraduate, and postgraduate education. To ensure compliance with this inclusion criterion, a screening question was included at the beginning of the questionnaire. Participants were required to purchase or consume organic products during the month preceding the survey, because the primary objective of the study was to explain actual organic food purchasing behavior rather than hypothetical purchase intentions. Restricting the sample to consumers with recent purchasing experience reduced recall bias and ensured that participants evaluated the proposed constructs based on relatively recent consumption experiences rather than abstract perceptions or intentions. Data were collected between March and May 2026.

The questionnaire was administered online using Google Forms and disseminated through commonly used social media platforms, including Facebook, Instagram, WhatsApp, and LinkedIn. To ensure methodological consistency, we applied the same recruitment strategy across Ecuador, Peru, and Chile. No country-specific recruitment procedures or incentives were implemented, which allowed participants from the three countries to be recruited under comparable conditions. To ensure data quality, the database was reviewed before statistical analyses were conducted. The responses were screened to identify incomplete questionnaires, duplicate submissions, and potential careless response patterns. In addition, response consistency was examined to detect straight-lining behavior, particularly when participants selected the same response category across all measurement items. Cases with incomplete information or clearly inconsistent response patterns were excluded from the final dataset.

### 3.2. Statistical Procedure

The study sample consisted of 766 participants: 310 Ecuadorians, 259 Peruvians, and 197 Chileans. Following the methodological guidelines proposed by Leguina [106], both measurement and structural models were evaluated. Furthermore, given that data were collected from three different countries, the recommendations of Henseler et al. [107] were followed by applying the Measurement Invariance of Composite Models (MICOM) procedure and conducting a Multigroup Analysis (MGA). Accordingly, the statistical analyses were conducted in three stages.

Phase 1: The measurement model was used to verify the psychometric properties of the constructs included in the study. First, the individual factor loading of each indicator was examined as preliminary evidence of convergent validity. Subsequently, internal consistency reliability was evaluated using Cronbach’s alpha and composite reliability coefficients (Rho_A and Rho_C), with values above 0.70 indicating satisfactory reliability. Convergent validity was further assessed through the Average Variance Extracted (AVE), for which values exceeding 0.50 were considered acceptable [108]. Discriminant validity was examined using the Fornell–Larcker criterion, which requires the square root of the AVE of each construct to exceed its correlations with all other constructs in the model [109]. In addition, the heterotrait-monotrait ratio (HTMT) was employed, with values below the recommended threshold of 0.90, indicating adequate conceptual distinctiveness among the constructs [110].

Phase 2: The Measurement Invariance of Composite Models (MICOM) procedure was applied to verify whether the constructs were measured equivalently across all countries. Establishing measurement invariance is a prerequisite for meaningful group comparisons because it ensures that any observed differences are attributable to substantive phenomena rather than measurement inconsistencies [107]. Subsequently, a permutation-based Multigroup Analysis (MGA) was conducted to determine whether the structural relationships proposed in the model differed significantly across national contexts, thereby assessing the robustness and generalizability of the findings across countries [111].

Phase 3: The structural model was evaluated using partial least-squares structural equation modeling (PLS-SEM). The objective of this stage is to examine the causal relationships proposed in the conceptual framework and empirically test the formulated hypotheses. The analysis included the estimation of standardized path coefficients (β) and assessment of their statistical significance, allowing for the determination of both the magnitude and direction of the relationships among the constructs. Furthermore, the overall model fit was assessed using the Standardized Root Mean Square Residual (SRMR), with values below 0.08 indicating an acceptable fit. Finally, the explanatory power of the model was evaluated through the coefficients of determination (R^2^), adopting values greater than 0.10 as the minimum acceptable threshold, in accordance with widely recognized methodological recommendations in the specialized literature [110,111,112,113].

## 4. Results

### 4.1. Demographic Analysis

The demographic profile of the sample reveals considerable geographic diversity, with participants primarily drawn from Ecuador (40.5%), followed by Peru (33.8%) and Chile (25.7%). This distribution provides an adequate representation of the three Latin American contexts characterized by distinct sociocultural backgrounds. In terms of gender, the sample exhibited a relatively balanced composition, comprising men (50.1%), women (47.7%), and non-binary individuals (2.2%), thereby ensuring the inclusion of diverse gender identities in the analysis. Regarding educational attainment, the sample displayed a comparatively high educational profile, with university graduates representing the largest segment (31.2%), followed by individuals with technical education (24.8%), secondary education (22.2%), and postgraduate qualifications (21.8%). This composition suggests that respondents possessed sufficient educational capital to understand and evaluate information related to sustainability and responsible consumption. Concerning age distribution, the sample is characterized by a strong presence of younger generational cohorts. Centennials constituted the largest group (33.9%), followed by Younger Millennials (20.8%), indicating that more than half of the participants belonged to relatively young consumer segments. Smaller proportions correspond to Mid Millennials (15.0%) and Older Millennials (15.7%), while older age groups are less represented, including Generation X (6.8%) and Baby Boomers (7.8%). Overall, this demographic profile suggests a predominance of individuals who have grown up in highly digitalized environments and have been extensively exposed to social media platforms and digital interactions (see Table 1).

### 4.2. Evaluation of the Measurement Model (Convergent and Discriminant Validity)

Following the guidelines proposed by Hair et al. [109] for evaluating measurement models, the convergent validity of the model was assessed by examining Cronbach’s Alpha, Composite Reliability (rho_a and rho_c), and Average Variance Extracted (AVE). According to the literature, a measurement model demonstrates adequate reliability and convergent validity when Cronbach’s Alpha and Composite Reliability values exceed 0.70; when the AVE is greater than 0.50, it remains lower than the corresponding Composite Reliability values [108]. To verify that these criteria were satisfied for each national sample (Ecuador: n = 310, Peru: n = 259, and Chile: n = 197) as well as for the overall model (n = 766), Table 2 presents the results of the aforementioned analyses separately for each country and for the pooled sample.

Discriminant validity was assessed using two complementary procedures: the Fornell–Larcker criterion and the Heterotrait-monotrait ratio (HTMT). Regarding the first procedure, the Fornell–Larcker criterion [109] was applied, which states that the square root of the AVE values reported on the diagonal of the matrix must exceed the correlations between the corresponding construct and all other constructs in the model. Compliance with this criterion indicates that each construct is more strongly explained by its own indicators than by the indicators associated with other constructs, thereby confirming that each variable adequately measures its intended theoretical concept. Concerning the second procedure, the HTMT ratio establishes that the relationships between each pair of constructs should remain below the threshold value of 0.90, demonstrating that the constructs are conceptually distinct and do not exhibit problematic overlap [110,112]. To verify that both criteria were satisfied for the overall sample (n = 766), as well as for each national subsample (Ecuador: n = 310, Peru: n = 259, and Chile: n = 197), Table 3 presents the results of the discriminant, HTMT (above the diagonal), and Fornell–Larcker (below the diagonal).

### 4.3. Common Method Bias

As all constructs were measured using self-reported questionnaires administered at a single point in time, several procedural and statistical remedies were implemented to minimize the potential influence of Common Method Bias (CMB). From a procedural perspective, participants were informed that their responses would remain anonymous and confidential, participation was entirely voluntary, and no right or wrong answers existed, thereby reducing their evaluation apprehension and social desirability bias. Harman’s single-factor test was conducted from a statistical perspective. The results indicated that the first unrotated factor explained less than 50% of the total variance, suggesting that common method variance was unlikely to represent a serious concern in this study. Furthermore, the full collinearity assessment showed Variance Inflation Factor (VIF) values below the recommended threshold of 3.3, providing additional evidence that common method bias did not substantially affect the results [111].

### 4.4. Multigroup Analysis

Given that the study combined data from Ecuador, Peru, and Chile, measurement invariance was assessed prior to conducting cross-country comparisons. Following the recommendations of Henseler et al. [107], the measurement invariance of the composite model (MICOM) procedure was implemented using SmartPLS 4. This procedure evaluates three sequential stages: (a) configural invariance, (b) compositional invariance, and (c) equality of the composite means and variances. Establishing at least partial measurement invariance is a prerequisite for conducting meaningful multi-group comparisons in PLS-SEM [107,111].

The results confirmed the compositional invariance for all constructs across the three country pairs (Ecuador–Peru, Ecuador–Chile, and Peru–Chile). Specifically, all original correlations exceeded the corresponding 5% quantiles of the permutation distribution and all permutation *p*-values were above the recommended threshold of 0.05. These findings indicate that the latent constructs were measured equivalently across the three national contexts, thereby satisfying the requirement for partial measurement invariance.

Subsequently, the equality of the composite means and variances was examined. Full measurement invariance was established for the Ecuador–Peru and Ecuador–Chile comparisons as no significant differences were detected in either composite means or variances (*p* > 0.05). For the Peru–Chile comparison, equality of means was confirmed for all constructs, whereas the EAT construct exhibited a significant difference in variance (*p* = 0.024). Nevertheless, because compositional invariance was achieved, partial measurement invariance was confirmed for all country pairs, which was sufficient to proceed with multigroup analysis [107] (See Table 4).

After establishing measurement invariance, permutation-based Multigroup Analysis (MGA) was conducted to examine whether the structural relationships differed across countries. The results revealed no statistically significant differences in any of the structural paths among Ecuador, Peru, and Chile (*p* > 0.05). Therefore, the relationships linking SMC, OMGS, EAT, GSI, and PBOP operate similarly across the three Latin American countries. These findings provide evidence for the robustness, stability, and cross-national applicability of the proposed SOR framework (see Table 5).

The primary contribution of this study is the cross-national validation of the proposed research model. The MICOM and MGA results indicate that the relationships between SMC, OMGS, EAT, GSI, and PBOP are statistically equivalent across Ecuador, Peru, and Chile. This finding suggests that the underlying psychological mechanisms proposed by the SOR framework are robust across Latin American contexts. Consequently, the influence of digital social interactions and environmental cognition on organic consumption appears to transcend national boundaries, providing evidence for the external validity and generalizability of the proposed model.

### 4.5. Evaluation of the Structural Model (Model Fitting and Hypothesis Testing)

The model was analyzed using the SmartPLS 4 software. To assess the causal relationships proposed in the model, the bootstrapping procedure with 5000 subsamples was applied [110]. The predictive power of the structural model was evaluated through the coefficients of determination (R^2^), with values exceeding 0.10 considered indicative of adequate explanatory power [112]. In addition, the Standardized Root Mean Square Residual (SRMR) was calculated to assess the average magnitude of the discrepancies between the observed and model-implied correlations, with values below 0.08, indicating an acceptable model fit [113]. Finally, the proposed hypotheses 1re evaluated for both the overall sample and each country-specific model by examining their statistical significance levels. Path coefficients with *p*-values below 0.05 were considered statistically significant, thereby providing empirical support for the validity of the hypothesized relationships. Table 6 presents the results of the aforementioned analyses.

## 5. Discussion

Having established the cross-national validity of the proposed research model and determined the acceptance or rejection of the hypothesized relationships, this study proceeds to discuss the findings. This section critically examines the results obtained and compares them with previous evidence reported in the literature in this field of research. To facilitate the interpretation of the findings, the discussion is organized around the research questions presented in the introduction, thereby providing a structured explanation of how the results contribute to addressing the study’s primary research objectives. It is important to note that the proposed relationships should be interpreted as theoretical associations rather than as evidence of temporal or causal development. Although the SOR framework provides a sequential conceptual explanation linking digital stimuli, psychological mechanisms, and purchasing behavior, the cross-sectional nature of the data does not permit causal inferences. Therefore, the findings should be understood to support the proposed theoretical sequence and demonstrate associations consistent with the hypothesized relationships.

### 5.1. Influence of SMC on EAT and GSI of South American Consumers

Regarding H1a, the results indicate that SMC consistently influences the EAT of South American consumers, as the hypothesis was supported in both the Overall Model (β = 0.318; *p* < 0.05) and country-specific models for Ecuador (β = 0.359; *p* < 0.05), Peru (β = 0.295; *p* < 0.05), and Chile (β = 0.282; *p* < 0.05). This finding confirms that exposure to posts, images, videos, programs, and social media accounts focused on environmental protection contributes to the development of positive evaluations and attitudes toward environmental conservation. This result is consistent with previous research that conceptualizes SMC as a communication resource capable of delivering relevant, persuasive, and visually engaging information [49]. Furthermore, the influence of such content depends on its perceived usefulness, credibility, and relevance to consumers [51]. Prior studies have shown that exposure to digital content can strengthen cognitive frameworks that support sustainability [57], whereas sustainability-related content can reinforce favorable attitudes toward environmentally responsible alternatives [58,59]. Therefore, the influence of SMC on EAT operates primarily at an informational and evaluative level, helping consumers recognize, appreciate, and legitimize the environmental benefits associated with pro-environmental behaviors.

Regarding H1b, the results revealed a more complex and theoretically meaningful pattern. SMC did not exert a significant influence on GSI in the Overall Model (β = 0.044; *p* > 0.05), Ecuador (β = 0.062; *p* > 0.05), or Peru (β = 0.024; *p* > 0.05), although a significant positive effect was observed in Chile (β = 0.112; *p* < 0.05). This interesting finding is that the direct relationship between SMC and GSI was not supported in the overall sample but became significant among Chilean consumers. One possible explanation is that the Chilean context provides a more favorable environment for the internalization of sustainability-related digital content into consumers’ self-concept. This finding suggests that environmental content disseminated through social media may be sufficient to shape attitudes but is not necessarily capable of leading consumers to incorporate sustainability into their self-concept. In this regard, the results partially align with the critical literature, indicating that the effects of digital content on sustainable behavior are neither automatic nor linear, as understanding of the psychological mechanisms through which digital influences are transformed into deeper internal responses remains fragmented [54,60,61]. These findings demonstrate that, while social media can promote environmental engagement, such engagement does not necessarily translate into a stable identity-based internalization of sustainability. Nevertheless, previous research has shown that social media can contribute to the construction of meanings, values, and sustainability-oriented perspectives [57], which may help explain the significant effect observed in Chile. From this perspective, the Chilean case may reflect sociocultural conditions that are conducive to transforming environmental content into personal identification with green values. However, the rejection of H1b in the overall model as well as in Ecuador and Peru suggests that GSI requires more intensive processes of internalization, personal experience, social validation, and behavioral consistency than can be achieved through mere exposure to digital content alone.

### 5.2. Influence of OMGS on EAT and GSI of South American Consumers

The results obtained for H2a indicate that OMGS constitutes a relevant antecedent of EAT in the overall model, as well as in Peru and Chile, but not in Ecuador. In the overall model of South American consumers, the relationship was positive and statistically significant (β = 0.340; *p* < 0.05), demonstrating that support received from digital communities focused on environmental issues contributes to strengthening favorable evaluations of sustainability and organic products. This finding is consistent with prior research suggesting that online communities provide informational, emotional, and social support capable of shaping consumers’ perceptions and orientations [36], while interactions with individuals who share environmental values facilitate the development of beliefs and norms that support sustainable behaviors [63,64]. The results observed in Peru (β = 0.287; *p* < 0.05) and Chile (β = 0.370; *p* < 0.05) further reinforce this perspective, suggesting that social validation, experience sharing, and a sense of belonging fostered within digital communities function as effective mechanisms for strengthening EAT. However, the rejection of this hypothesis in Ecuador (β = 0.355; *p* > 0.05) introduces an important nuance. Although Ecuadorian consumers receive support and engage in interactions within virtual communities, such support does not appear sufficient to directly modify their environmental evaluations, reflecting greater heterogeneity in how Ecuadorian consumers use online communities when forming environmental evaluations or the presence of other relevant influences, such as personal experiences, offline social norms, or institutional information. Therefore, the finding should not be interpreted as contradicting the proposed relationship but rather as evidence that the effect of online member group support on environmental attitudes may be more context-dependent among Ecuadorian consumers. This finding may be interpreted in light of the argument that digital social influence does not always translate automatically into attitudinal change, as consumers may process environmental information based more on individual criteria than on collective influence [31]. Consequently, the findings suggest that online social support exerts a meaningful influence on EAT across most of the contexts examined, although its effectiveness depends on the sociocultural conditions under which individuals interpret and evaluate interactions originating from their digital communities.

The results for H2b reveal a pattern that differs from that observed for H2a and provides particularly interesting evidence regarding the formation of GSI. Hypothesis H2b was supported in the overall model (β = 0.082; *p* < 0.05) and in Ecuador (β = 0.094; *p* < 0.05) but not in Peru (β = 0.044; *p* > 0.05) or Chile (β = 0.118; *p* > 0.05). These findings indicate that support received from digital communities can contribute to strengthening GSI when the South American sample is considered as a whole; however, its capacity to generate a stable ecological identity is inconsistent across national contexts. From a theoretical perspective, this finding is supported by evidence suggesting that perceived social support in digital environments can strengthen individuals’ self-perceptions by providing validation, recognition, and reinforcement of shared behaviors [61]. Moreover, continuous interaction with individuals who endorse similar environmental values may facilitate the incorporation of such values into their self-concept [63,65]. This reasoning helps explain why the relationship is significant in the overall model, particularly in Ecuador, where digital communities appear to function as spaces of social legitimization that reinforces the perception of being an environmentally responsible consumer. Nevertheless, rejection of the hypothesis in Peru and Chile suggests that GSI represents a deeper psychological structure that does not depend exclusively on the approval or support received through social media. Consequently, although online communities may foster a sense of belonging and provide emotional support, such stimuli are not always sufficient for consumers to internalize a stable green identity. Overall, the findings indicate that OMGS possess only a limited capacity to consistently shape GSI across countries, suggesting that identity formation is influenced by more complex and enduring factors than mere participation in digital communities.

### 5.3. Influence of EAT on GSI of South American Consumers

The results obtained for H3a indicate that EAT is one of the most important antecedents of GSI among South American consumers. This hypothesis was supported in the overall model (β = 0.516; *p* < 0.05), Ecuador (β = 0.516; *p* < 0.05), Peru (β = 0.460; *p* < 0.05), and Chile (β = 0.586; *p* < 0.05), demonstrating remarkable consistency across national contexts. These findings indicate that, as consumers develop more favorable evaluations of environmental protection and organic products, they are also more likely to incorporate such values into their personal self-concept. From a theoretical perspective, this result supports the proposition that EAT functions as a motivational mechanism that guides individuals toward lifestyles aligned with sustainability principles [93,97,98]. Likewise, the findings are consistent with green identity literature, which argues that environmental identity emerges when ecological concerns evolve beyond simple cognitive evaluations and become integrated into an individual’s self-definition [16,17,91].

The fact that this relationship is significant across all three countries suggests that the transition from attitude to identity represents a relatively universal process in the South American context. Furthermore, the stronger effect observed in Chile may indicate greater integration of environmental values into the self-concept of Chilean consumers. Taken together, these results confirm that EAT functions not only as a favorable evaluation of environmental issues but also as a key psychological mechanism through which a durable GSI is formed. In so doing, the findings provide strong empirical support for one of the central propositions of the SOR framework.

### 5.4. Mediating Effect of EAT on the Relationship Between SMC, OMGS, and GSI of South American Consumers

The results for H3b demonstrated that EAT played a significant mediating role in the relationship between SMC and GSI in the overall model (β = 0.164; *p* < 0.05), Ecuador (β = 0.186; *p* < 0.05), Peru (β = 0.136; *p* < 0.05), and Chile (β = 0.165; *p* < 0.05). This finding is particularly noteworthy considering that the direct relationship between SMC and GSI was not supported in the overall model in Ecuador and Peru. This suggests that content disseminated through social media does not strengthen green identity directly but rather through the prior development of favorable EAT. From the perspective of the SOR framework, these results confirm that digital stimuli must first be internalized through cognitive and evaluative processes before being integrated into an individual’s self-concept. This interpretation is consistent with prior research indicating that environmental content on social media contributes to the development of cognitive frameworks that support sustainability [53] and that digital content can strengthen favorable orientations toward environmentally responsible consumption [52]. Likewise, the findings support previous arguments that the influence of digital content on sustainable behaviors and orientations often operates through intermediate psychological mechanisms [54,60]. Consequently, the observed mediation effect indicates that GSI does not emerge directly from exposure to environmental messages; rather, it develops when such messages first alter consumers’ environmental evaluations and beliefs. This finding helps explain why the direct relationship between SMC and GSI yielded inconsistent results across countries, whereas the indirect effect through EAT proved robust and significant in all the contexts examined.

The results for H3c reveal that EAT significantly mediated the relationship between OMGS and GSI in the overall model (β = 0.175; *p* < 0.05), Ecuador (β = 0.183; *p* < 0.05), Peru (β = 0.132; *p* < 0.05), and Chile (β = 0.217; *p* < 0.05). This finding indicates that support received from virtual communities focused on sustainability does not strengthen GSI solely through social interaction itself; rather, such interaction first contributes to the development of favorable EAT. These results are consistent with studies suggesting that digital communities provide informational and emotional resources capable of shaping consumer perceptions [36] and that interactions with individuals who share environmental values facilitate the consolidation of ecological beliefs and orientations [63,65]. However, the present findings extend this body of knowledge by demonstrating that EAT serves as an explanatory mechanism linking social support to the development of GSI. From a critical perspective, this result helps explain why the direct relationship between OMGS and GSI was inconsistent across countries and was significant only in Ecuador. The observed mediation suggests that digital communities do not automatically generate an ecological identity; instead, they first strengthen EAT, which is subsequently internalized by individuals as part of their self-concept. Furthermore, the stronger effect observed in Chile suggests that Chilean consumers may possess a greater capacity to transform digital social influence into environmental identification processes. Taken together, these findings reinforce the sequential logic proposed by the SOR framework and confirm that EAT constitutes the primary psychological mechanism by which both communicative and social stimuli are transformed into a consolidated GSI.

Overall, the mediation analysis revealed that the role of environmental attitudes is not uniform across the three national contexts. In some country-specific models, environmental attitude operates as a full mediator, indicating that the influence of digital stimuli is transmitted entirely through consumers’ environmental evaluations. In contrast, other models exhibited partial mediation, suggesting that digital stimuli retained a direct influence alongside an indirect pathway through environmental attitudes. These differences highlight that the psychological mechanisms linking social media environments with green self-identity are context-dependent and may vary according to the characteristics of each national setting, reinforcing the importance of examining mediation effects through cross-country analyses, rather than assuming a homogeneous behavioral process.

### 5.5. Influence of GSI on PBOP of South American Consumers

The results obtained for H4 confirm that GSI is one of the strongest determinants of PBOP among South American consumers. This hypothesis was supported in the overall model (β = 0.554; *p* < 0.05), Ecuador (β = 0.559; *p* < 0.05), Peru (β = 0.521; *p* < 0.05), and Chile (β = 0.602; *p* < 0.05), demonstrating the remarkable stability of the relationship across the three national contexts examined. These findings indicate that when individuals incorporate environmental values into their self-concept and perceive themselves as consumers committed to sustainability, the likelihood that such convictions translate into actual organic food-purchasing decisions increases substantially. From a theoretical perspective, the results support the proposition that environmental identity promotes consistency between personal values and observable behaviors [91]. Likewise, they are consistent with previous studies suggesting that GSI strengthens consumers’ willingness to engage in behaviors aligned with environmental protection [13,14,15,16,17]. Furthermore, the findings corroborate more recent research identifying GSI as a mechanism capable of transforming environmental concerns into concrete marketplace actions, thereby reinforcing the persistence of sustainable behaviors over time [93,97,98]. From a critical standpoint, the magnitude of the coefficients observed across all three countries suggests that GSI possesses greater explanatory power than any other antecedent examined in the model, including digital and social stimuli. While SMC and OMGS exhibited heterogeneous effects across countries, the influence of GSI on PBOP remained consistently strong in all the contexts analyzed. Chile exhibited the strongest effect, which may reflect a greater integration of environmental values into consumers’ purchasing patterns. Taken together, these findings confirm one of the central propositions of sustainable consumption literature: consumers do not purchase organic products solely because they are exposed to environmental information or receive social support; rather, such influences become effective when they are internalized as part of who consumers are. Therefore, GSI emerges as the final psychological mechanism connecting environmental and social influences with the actual enactment of sustainable purchasing behavior, establishing itself as the most influential variable in explaining PBOP in the South American context.

## 6. Conclusions

The findings of the present study indicate that both SMC and the support provided by OMGS play significant roles in shaping EAT among PBOP in South America. Overall, the results demonstrate that digital stimuli primarily influence EAT, confirming that exposure to information, messages, experiences, and discussions related to sustainability fosters favorable evaluations of environmental protection and responsible consumption. However, the findings also revealed that the capacity of these stimuli to directly strengthen GSI is limited and context-dependent, suggesting that the development of an ecological identity constitutes a more complex psychological process than the mere reception of information or engagement in digital social interactions.

One of the most important contributions of this study is that it demonstrates that EAT serves as the central mechanism through which digital influences are internalized by consumers. The results showed that GSI does not emerge directly from communicational or social stimuli; rather, it develops when such stimuli first transform individuals’ environmental evaluations. In this regard, the EAT functions as a psychological bridge that converts digital influence into deeper ecological identification processes. This evidence reinforces the sequential logic proposed by the SOR framework by confirming that consumers do not respond automatically to stimuli originating from the digital environment. Instead, they cognitively process and interpret these influences before integrating them into their beliefs and values.

Furthermore, the findings indicate that GSI is the primary antecedent of PBOP. Beyond the influence of SMC or the support received through OMGS, consumers purchase organic products primarily when such behavior is perceived as consistent with their self-concept. This finding suggests that sustainable purchasing decisions are not determined exclusively by informational or social factors but rather by individuals’ capacity to integrate environmental values into their identity. Consequently, GSI has emerged as the key psychological mechanism linking external influences to the actual enactment of sustainable consumption behaviors.

From a comparative perspective, the results reveal substantial similarities across Ecuador, Peru, and Chile regarding the manner in which SMC and OMGS influence EAT and how these attitudes subsequently contribute to the formation of GSI and PBOP. Nevertheless, differences were observed in the magnitude and significance of certain direct relationships, particularly those associated with the development of an ecological identity. These variations suggest that the internalization of environmental values may be shaped by sociocultural conditions, institutional environments, and the maturity of the sustainable markets within each country. Therefore, although the proposed model demonstrates strong explanatory power across all three contexts, the findings also indicate that the influence of digital stimuli on GSI is not uniform throughout the region.

From a theoretical standpoint, this study contributes to the sustainable consumption literature by demonstrating that EAT and GSI perform distinct functions within the consumer decision-making process. While the EAT represents a cognitive and evaluative response that is relatively sensitive to digital stimuli, the GSI constitutes a deeper and more stable psychological structure that requires higher levels of internalization. This distinction helps explain why many environmental communication strategies succeed in raising consumer awareness, but do not necessarily generate enduring changes in consumption patterns. Consequently, this study advances the understanding of the mechanisms underlying the transition from digital influence to the effective adoption of PBOP, while providing valuable empirical evidence from the Latin American context.

Finally, although country-specific analyses revealed descriptive differences in the magnitude and statistical significance of several structural relationships, these variations should be interpreted with caution. The multigroup analysis did not identify statistically significant differences in the structural relationships between Ecuador, Peru, and Chile, suggesting that the proposed SOR framework operates in a broadly comparable manner across the three national contexts. Consequently, the observed country-specific variations are better understood as descriptive differences in the strength or statistical significance of individual paths, rather than evidence of genuine structural heterogeneity. This finding reinforces the robustness and cross-context applicability of the proposed theoretical model while highlighting the importance of distinguishing descriptive comparisons from formal statistical evidence when interpreting cross-country analyses.

## 7. Theoretical, Practical and Social Implications

### 7.1. Theoretical Implications

This study contributes to the literature on sustainable consumption by strengthening and refining the explanatory power of the SOR framework within the context of PBOP in South America. These findings confirm that SMC and OMGS operate as external stimuli, while EAT and GSI function as organismic states that transform these influences into PBOP. More importantly, the results demonstrated that not all internal psychological mechanisms respond to digital stimuli in the same manner. The EAT emerged as a more immediate and stimulus-sensitive construct, whereas the GSI represented a deeper level of internalization that required prior attitudinal development. The significant mediating role of EAT further reinforces the sequential logic proposed by the SOR framework, revealing that digital influences do not directly generate ecological identities, but instead operate through a process of cognitive and evaluative transformation. Consequently, this study extends the existing theory by providing empirical evidence that sustainable consumer behavior is the outcome of a progressive psychological process that begins with digital stimuli, evolves through attitude formation, and culminates in identity-driven purchasing behavior.

### 7.2. Practical Implications

The findings of this study have several practical implications for stakeholders involved in promoting sustainable consumption. For policymakers, the results suggest that environmental policies should complement traditional awareness campaigns with digital communication strategies to strengthen both environmental attitudes and green self-identity. In this sense, environmental organizations may benefit from developing interactive online communities that facilitate information sharing, peer support, and active engagement with sustainability initiatives rather than relying solely on one-way communication. Likewise, social media managers should prioritize credible, educational, and emotionally engaging sustainability-related content that is capable of fostering meaningful consumer interaction and long-term value internalization. However, organic food producers are encouraged to integrate educational social media content with community-building strategies that promote consumer participation, trust, and lasting relationships beyond transactional marketing. Finally, sustainability educators and higher education institutions can leverage social media as a complementary learning environment by incorporating digital sustainability campaigns, collaborative online activities, and environmentally oriented communities that reinforce ecological values and encourage sustainable purchasing behaviors among the younger generations.

### 7.3. Social Implications

From a social perspective, this study demonstrates that sustainable consumption is not determined solely by environmental knowledge but rather by the extent to which individuals internalize environmental values through attitude formation and identity development. This finding provides important insights for governments, educational institutions, and environmental organizations seeking to promote sustainable lifestyles. The results suggest that public policies and environmental communication campaigns should move beyond the mere dissemination of information and instead focus on fostering favorable EAT and strengthening GSI among citizens. Social media platforms and digital communities have emerged as strategic tools to enhance environmental literacy, encourage social participation, and reinforce collective commitment to sustainability. Moreover, the cross-national evidence obtained from Ecuador, Peru, and Chile indicates that, although common psychological mechanisms operate across South America, environmental communication initiatives should also consider local sociocultural characteristics to maximize their effectiveness and support the long-term adoption of sustainable consumption practices.

## 8. Limitations and Recommendations for Future Research

Although this study provides valuable insights into the mechanisms linking digital social environments with PBOP, several limitations should be acknowledged. First, its cross-sectional design precludes causal inferences and captures consumer perceptions at a single point in time. Future studies could employ longitudinal designs to examine how environmental attitudes and green self-identity evolve over time, and whether these psychological mechanisms remain stable. Second, all variables were measured using self-reported questionnaires, which may be affected by common method variance and social desirability bias, despite the procedural and statistical measures adopted to mitigate these concerns. Future research could combine survey data with behavioral or observational measures to enhance measurement validity. Third, convenience sampling limits the statistical generalizability of the findings; therefore, probability-based sampling strategies involving more heterogeneous populations are encouraged. Fourth, this study was conducted exclusively in Ecuador, Peru, and Chile. Although these countries provide an appropriate setting for cross-country validation within comparable Latin American contexts, future investigations should examine the proposed model in culturally distinct regions to assess its cross-cultural robustness. Finally, the sample was restricted to consumers who had recently purchased organic products, excluding environmentally concerned individuals who had not translated their attitudes into actual purchasing behavior. Future research could compare organic consumers with non-purchasers to better understand the attitude–behavior gap. Additionally, experimental studies could evaluate the causal effects of different types of sustainability-related social media content, whereas qualitative approaches may provide deeper insights into how consumers internalize digital environmental messages and develop green self-identity across diverse sociocultural contexts.

## Figures and Tables

**Table 1:** Demographic results (n = 766).

Variable		f	%
Country	Ecuador	310	40.5%
	Perú	259	33.8%
	Chile	197	25.7%
Gender	Male	384	50.1%
	Female	365	47.7%
	Non-binary	17	2.2%
Education level	Postgraduate	167	21.8%
	Graduate	239	31.2%
	School technician	190	24.8%
	Secondary	170	22.2%
Age	After 2000 (Centennials)	260	33.9%
	1995 to 2000 (Younger millennials)	159	20.8%
	1994 to 1989 (Mid Millennials)	115	15.0%
	1988 to 1979 (Older Millennials)	120	15.7%
	1978 to1965 (Generation X)	52	6.8%
	Before 1965 (Baby Boomers)	60	7.8%

**Table 2:** Convergent Validity.

Model/Country	Test Applied	SMC	OMGS	EAT	GSI	PBOP
Overall (n = 766)	Cronbach	0.840	0.835	0.857	0.851	0.861
	rho_a	0.842	0.837	0.860	0.853	0.864
	rho_c	0.893	0.890	0.903	0.900	0.906
	AVE	0.676	0.670	0.700	0.692	0.706
Ecuador (n = 310)	Cronbach	0.863	0.828	0.867	0.848	0.868
	rho_a	0.871	0.832	0.867	0.849	0.877
	rho_c	0.906	0.886	0.909	0.898	0.910
	AVE	0.708	0.660	0.714	0.687	0.716
Perú (n = 259)	Cronbach	0.801	0.838	0.827	0.842	0.849
	rho_a	0.811	0.840	0.832	0.848	0.851
	rho_c	0.870	0.891	0.885	0.894	0.898
	AVE	0.626	0.672	0.658	0.678	0.688
Chile (n = 197)	Cronbach	0.848	0.843	0.872	0.868	0.866
	rho_a	0.855	0.843	0.882	0.870	0.869
	rho_c	0.897	0.895	0.912	0.910	0.909
	AVE	0.686	0.680	0.721	0.717	0.713

**Table 3:** Discriminant validity (Fornell–Larcker and HTMT).

**Fornell–Larcker and HTMT Test for the Overall Model (n = 766)**					
	SMC	OMGS	EAT	GSI	PBOP
SMC	0.822	0.277	0.467	0.212	0.164
OMGS	0.233	0.818	0.487	0.338	0.220
EAT	0.398	0.414	0.837	0.622	0.412
GSI	0.181	0.286	0.533	0.832	0.645
PBOP	0.142	0.187	0.355	0.554	0.840
**Fornell–Larcker and HTMT test: Ecuador (n = 310)**					
	SMC	OMGS	EAT	GSI	PBOP
SMC	0.841	0.291	0.512	0.221	0.196
OMGS	0.250	0.812	0.522	0.367	0.294
EAT	0.448	0.444	0.845	0.618	0.434
GSI	0.193	0.308	0.531	0.829	0.645
PBOP	0.178	0.249	0.377	0.559	0.846
**Fornell–Larcker and HTMT test: Peru (n = 259)**					
	SMC	OMGS	EAT	GSI	PBOP
SMC	0.791	0.134	0.388	0.214	0.173
OMGS	0.102	0.820	0.380	0.227	0.172
EAT	0.324	0.317	0.811	0.573	0.329
GSI	0.178	0.193	0.482	0.823	0.612
PBOP	0.144	0.146	0.277	0.521	0.829
**Fornell–Larcker and HTMT test: Chile (n = 197)**					
	SMC	OMGS	EAT	GSI	PBOP
SMC	0.828	0.413	0.474	0.195	0.102
OMGS	0.351	0.825	0.538	0.413	0.162
EAT	0.412	0.469	0.849	0.677	0.469
GSI	0.170	0.353	0.595	0.847	0.692
PBOP	0.089	0.139	0.412	0.602	0.845

**Table 4:** MICOM results across country pairs.

Comparison	Step 2: Compositional Invariance	Step 3a: Equality of Means	Step 3b: Equality of Variances	Conclusion
Ecuador–Perú	Supported	Supported	Supported	Full Measurement Invariance
Ecuador–Chile	Supported	Supported	Supported	Full Measurement Invariance
Chile–Perú	Supported	Supported	Partially Supported *	Partial Measurement Invariance

* EAT showed a significant variance difference (*p* = 0.024).

**Table 5:** MGA (permutation-based MGA results).

Structural Path	Ecuador–Perú (*p*-Value)	Ecuador–Chile (*p*-Value)	Perú–Chile (*p*-Value)
SMC–EAT	0.350	0.324	0.872
SMC–GSI	0.280	0.533	0.090
OMGS–EAT	0.323	0.836	0.311
OMGS–GSI	0.512	0.777	0.374
EAT–GSI	0.505	0.408	0.134
GSI–PBOP	0.492	0.483	0.201

Note: No statistically significant differences were identified between pairs of countries (all *p*-values > 0.05).

**Table 6:** Hypotheses testing.

**Overall Model. R^2^ EAT: 0.265; R^2^ GSI: 0.288; R^2^ PBOP: 0.306; SRMR: 0.046**				
**Hypotheses**	**Relation**	***β***	***p*-Value**	**Hypotheses**
H1a	SMC-EAT	0.318	0.000	Accepted
H1b	SMC-GSI	0.044	0.176	Rejected
H2a	OMGS-EAT	0.340	0.000	Accepted
H2b	OMGS-GSI	0.082	0.000	Accepted
H3a	EAT-GSI	0.516	0.000	Accepted
H3b	SMC-EAT-GSI	0.164	0.000	Accepted
H3c	OMGS-EAT-GSI	0.175	0.000	Accepted
H4	GSI-PBOP	0.554	0.000	Accepted
**Ecuador Model. R^2^ EAT: 0.314; R^2^ GSI: 0.284; R^2^ PBOP: 0.310; SRMR: 0.055**				
H1a	SMC-EAT	0.359	0.000	Accepted
H1b	SMC-GSI	0.062	0.242	Rejected
H2a	OMGS-EAT	0.355	0.076	Rejected
H2b	OMGS-GSI	0.094	0.000	Accepted
H3a	EAT-GSI	0.516	0.000	Accepted
H3b	SMC-EAT-GSI	0.186	0.000	Accepted
H3c	OMGS-EAT-GSI	0.183	0.000	Accepted
H4	GSI-PBOP	0.559	0.000	Accepted
**Perú Model: R^2^ EAT: 0.180; R^2^ GSI: 0.226; R^2^ PBOP: 0.268; SRMR: 0.056**				
H1a	SMC-EAT	0.295	0.000	Accepted
H1b	SMC-GSI	0.024	0.671	Rejected
H2a	OMGS-EAT	0.287	0.000	Accepted
H2b	OMGS-GSI	0.044	0.423	Rejected
H3a	EAT-GSI	0.460	0.000	Accepted
H3b	SMC-EAT-GSI	0.136	0.000	Accepted
H3c	OMGS-EAT-GSI	0.132	0.000	Accepted
H4	GSI-PBOP	0.521	0.000	Accepted
**Chile Model: R^2^ EAT: 0.283; R^2^ GSI: 0.361; R^2^ PBOP: 0.359; SRMR: 0.056**				
H1a	SMC-EAT	0.282	0.000	Accepted
H1b	SMC-GSI	0.112	0.044	Accepted
H2a	OMGS-EAT	0.370	0.000	Accepted
H2b	OMGS-GSI	0.118	0.055	Rejected
H3a	EAT-GSI	0.586	0.000	Accepted
H3b	SMC-EAT-GSI	0.165	0.000	Accepted
H3c	OMGS-EAT-GSI	0.217	0.000	Accepted
H4	GSI-PBOP	0.602	0.000	Accepted

Note: While the proposed model explains a substantial proportion of the variance in environmental attitudes and ecological self-identity, the reported R^2^ values also indicate that these constructs could be influenced by factors beyond those considered in this study. This highlights the need for this research model to be expanded by including other factors that may contribute to the generation of environmental attitudes and green self-identity.

## Data Availability

The data presented in this study are openly available at: https://drive.google.com/drive/folders/1minD62TN6lszq1fib2HbekwXwbOKjLA3?usp=sharing (accessed on 1 June 2026).

## Appendix A. Questions Used to Measure the Variables

**Var.**	**Items**
SMC [105]	1. I often read information and articles about environmental protection on social media.
	2. I often view images and videos about environmental protection on social media.
	3. I often watch programs or audiovisual content related to environmental protection on social media.
	4. I often follow accounts of organizations that promote sustainable lifestyles on social media.
OMGS [36]	1. My friends on social media show concern for the environment.
	2. My friends on social media share information about environmental care.
	3. My friends on social media give me advice when I ask them about topics related to environmental care.
	4. I receive interactions (likes or comments) when I share information about environmental care on social media.
GSI [95]	1. I consider myself a person concerned about environmental issues.
	2. I consider myself a consumer who prefers environmentally friendly products.
	3. I consider myself a consumer who feels good about choosing environmentally friendly products.
	4. I feel completely satisfied with myself for choosing environmentally friendly products.
EAT [1]	1. I believe that organic products help save nature and its resources.
	2. Environmental protection is important to me when purchasing products.
	3. I have a favorable attitude toward purchasing organic products.
	4. If I have the choice, I prefer an organic product to a conventional one.
GBP [1]	1. I regularly buy organic products.
	2. I consistently buy organic products for my daily needs.
	3. I have consistently bought organic products for the past six months.
	4. I buy organic products even when conventional alternatives are available.
